# Assessing yield gap in high productive countries by designing wheat ideotypes

**DOI:** 10.1038/s41598-019-40981-0

**Published:** 2019-04-02

**Authors:** Nimai Senapati, Mikhail A. Semenov

**Affiliations:** Department of Plant Sciences, Rothamsted Research, West Common, Harpenden, Herts AL5 2JQ United Kingdom

## Abstract

Designing crop ideotypes *in silico* is a powerful tool to explore the crop yield potential and yield gap. We defined yield gap as the difference between yield potential of a crop ideotype optimized under local environment and yield of an existing cultivar under optimal management. Wheat ideotypes were designed for the current climate using the Sirius model for both water-limited and irrigated conditions in two high wheat-productive countries viz. the United Kingdom (UK) and New Zealand (NZ) with the objective of estimating yield gap. The mean ideotype yields of 15.0–19.0 t ha^−1^ were achieved in water-limited conditions in the UK and NZ, whereas 15.6–19.5 t ha^−1^ under irrigated conditions. Substantial yield gaps were found in both water-limited, 28–31% (4–6 t ha^−1^), and irrigated conditions, 30–32% (5–6 t ha^−1^) in the UK and NZ. Both yield potential (25–27%) and yield gap (32–38%) were greater in NZ than the UK. Ideotype design is generic and could apply globally for estimating yield gap. Despite wheat breeding efforts, the considerable yield gap still potentially exists in high productive countries such as the UK and NZ. To accelerate breeding, wheat ideotypes can provide the key traits for wheat improvement and closing the yield gap.

## Introduction

To ensure food security for the world’s rapidly growing population, food production needs to increase substantially^1,2^. Demand for cereal is also expected to rise in coming decades for animal feed and production of beverages and bio-energy^3^. Wheat (*Triticum aestivum* L.) is one of the key staple crops in global food security, providing about 20% of total dietary calories and protein needs, with about 700 million tonnes of annual production from a harvested area of more than 220 million hectares globally^4,5^. With the limited scope for extending present crop-growing areas, a considerable increase in crop productivity is required to guarantee future food security^6,7^. When considering sustainable intensification, closing the yield gap could be essential for increasing crop productivity and food production towards food security^8,9^. Although a full yield gap closure is not feasible, economically viable, nor environmentally desirable, about 80% of yield potential is often assumed achievable^9,10^.

In general yield gap of a crop grown in a certain location and cropping system is defined as the difference between the potential yield of an adapted crop variety under irrigated or non-irrigated condition and average actual yield achieved by farmers^8–11^. Potential yield under irrigated condition is the yield of a crop cultivar when grown under optimal management practices with water and nutrients non-limiting and biotic stresses (disease, pest, weed etc.) effectively controlled^8–11^. Whereas, potential yield under non-irrigated or rainfed condition is the water-limited yield potential, i.e. yield of a crop cultivar limited by water, but not-limited by nutrients and biotic stresses^8–11^. Potential yield is usually estimated by using empirical and process-based simulation models, field experiments, yield contests, the highest yield records and 95-percentile of yield distributions^9,11–13^. However, achieving yield potential requires near perfect management of crop and soil factors along with coincidence of optimal climatic conditions that influence plant growth and development throughout the crop growth cycle^11,14^. Although a few superior farmers may come close to potential yield, it is not feasible for a large group of farmers to do so^11^. Thus, a gap always exists between the potential yield and the average farmer yield.

Yield gap analysis is important for (i) knowing and estimating the exploitable yield gap, (ii) increasing yield by knowing the factors that contribute the yield gap, (iii) motivation for increasing yield by knowing the gap and (iv) formulating policies and research priorities^9,11,12^. The main factors for yield gap are sub-optimal crop management practices, nutrient deficiency and imbalance, local soil problems (e.g., compaction, salinity, alkalinity, acidity, and Fe, Al or Bo toxicity), non-optimal sowing (timing or density), inferior seed quality, poor disease and pest control including weed, and abiotic stress (e.g., heat and water stress)^9,15,16^. In developed countries at high latitude, actual farmer yields are generally high and yield gaps are small mainly due to the favourable climatic conditions and availability of resources and advance technologies for optimal crop managements, for example, New Zealand (NZ) and north-western Europe including the United Kingdom (UK)^15,17,18^. Many studies estimated and reviewed yield gap for different cereal crops from field to regional and global scales^8–11,19^. However, the general yield gap idea does not account for potential genetic yield improvement^8–11^. In this study, we defined a yield gap (Y_G_) as the difference between ‘genetic’ yield potential under irrigated or non-irrigated condition and the management-optimal yield potential of a locally adapted current cultivar. ‘Genetic’ yield potential could be estimated by optimizing *in silico* physiological traits of crop ideotypes^6,20–25^.

The idea of ‘breeding of crop ideotypes’, in which breeders select plant ideotypes based on their knowledge of crop physiology for crop improvement in the target environment, and then breed for them, was first proposed by Donald^26^. A crop ideotype is a virtual idealized crop that is expected to produce a greater quality and quantity of grain yield when developed as a cultivar. Designing crop ideotypes and optimization of cultivar traits under target environments have gradually become a reality with the substantial increase in computational power of modern computers and the significant advances in process-based eco-physiological crop models^24,25,27,28^. These crop models are the most suitable and powerful tool for designing such crop ideotypes. Crop models help in a) designing crop ideotypes to estimate genetic yield potential, b) selecting optimal combination of target traits when considering possible trade-offs between them, c) assessing performance of potential candidate ideotypes across target environments^29,30^. Ideotype design together with the existence of a diverse natural genetic variations for cereal crops, for example for wheat, and recent advances in genomics and breeding technologies have high potentials for breeding of crop ideotypes by tapping the existing natural genetic variations to take the maximum advantages of local environments (e.g., climate)^3,6,20,31^. Thus, a yield gap, as assessed by designing ideotype, is exploitable and the gap could be narrowed down by crop improvement and genetic adaptation^3,6,7,18,32^.

Ideotype design for a target environment needs (i) a well-tested model and (ii) ideotype-optimization in a multidimensional cultivar parameter space to capture in full the parameter ranges and their possible interactions. In the present study, we designed wheat ideotypes using Sirius, a process-based crop model coupled with a powerful computational framework for designing wheat ideotype by utilizing the full parameter range in a multidimensional sapace^22,33,34^. Sirius is a well validated model for a range of modern wheat varieties under diverse climatic conditions across Europe including the United Kingdom (UK), New Zealand (NZ), Australia and the USA, including Free-Air CO_2_ Enrichment experiments^33–37^. In the present study, Sirius was used for designing wheat ideotypes under the local current climatic conditions across the UK and NZ.

The main objective of the present study was to estimate yield gap (Y_G_) of wheat in two high productive countries (the UK and NZ) by designing ideotypes.

## Results and Discussion

### Yield potential of wheat under current climate

**Figure 1:**
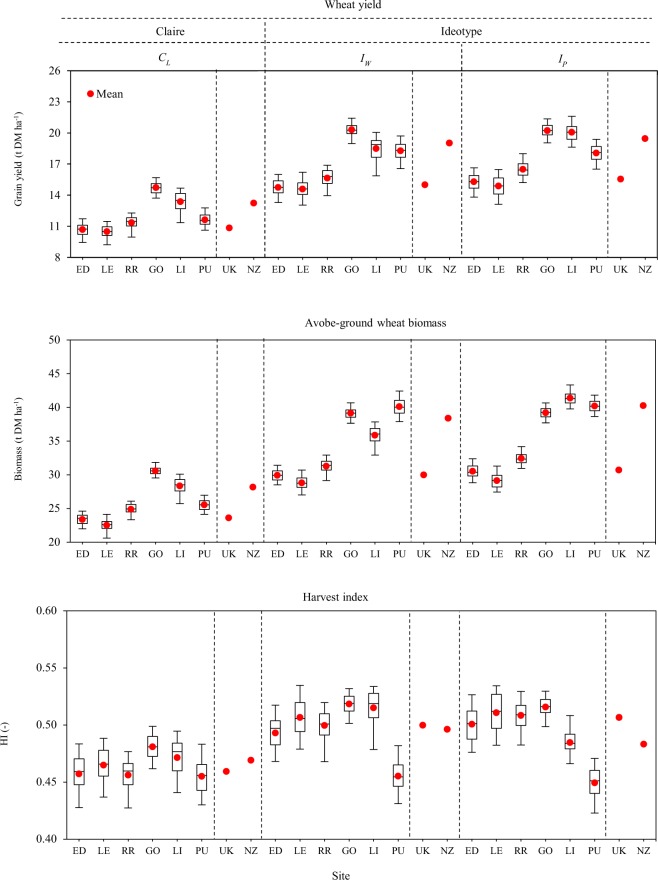
Grain yield, above-ground biomass and harvest index (HI) of locally adapted winter wheat *cv*. Claire (*C*_*L*_) under current climate, and wheat ideotypes optimized under current climate in water-limited (*I*_*W*_) and potential (*I*_*P*_) conditions. The box plots show 5, 25,50, 75 and 95-percentiles including mean. ED: Edinburgh, LE: Leeds, RR: Rothamsted, UK; GO: Gore, LI: Lincoln, PU: Pukekohe, NZ; UK: United Kingdom, NZ: New Zealand.

The Fig. 1 shows yield potentials of wheat ideotypes at three sites in each country viz. UK and NZ. The mean grain yields over three sites in each country for the ideotype *I*_*W*_, optimized under current climate in the water-limited (rainfed) condition, were 15.0 and 19.0 t ha^−1^ in the UK and NZ, respectively (Fig. 1). Whereas, averaged yields of the ideotype *I*_*P*_, optimized under the potential (irrigated) condition, were 15.6 and 19.5 t ha^−1^ in the UK and NZ, respectively. Mean yield variations (variance) between sites for both *I*_*W*_ and *I*_*P*_ were small (0.32~0.70 t ha^−1^) in UK, but greater (1.2~1.4 t ha^−1^) in NZ. The mean yield potentials of both ideotypes were 27% (4.0 t ha^−1^) and 25% (3.9 t ha^−1^) greater in NZ than the UK under water-limited and potential conditions, respectively (Fig. 1). Few farms have already achieved some record wheat yields (15~17 t ha^−1^) in recent years in the UK and NZ, for examples, 16.5 t ha^−1^ in Northumberland in the UK (2015) and 16.8 t ha^−1^ in Canterbury in NZ (2017)^38–41^. Craigie *et al*.^42^ obtained wheat yield of 15.9 t ha^−1^ in their experimental fields in NZ and believe that further increase is possible with the appropriate new cultivars. Mitchell and Sheehy^43^ have recently indicated that potential wheat yield could be 20 t ha^−1^ in most of the wheat growing region in the UK, using new wheat cultivars. The present study shows mean wheat yield potentials of 15–20 t ha^−1^ at national scales in the UK and NZ.

### Ideotypes traits optimized for maximize yield potential

#### Canopy architecture

The mean optimized potential maximum area of flag leaf (*A*_*Max*_) for *I*_*W*_ was 16% and 5% greater than baseline winter wheat *cv*. Claire (*C*_*L*_) in the UK and NZ respectively (Table 1). Further improvements in *A*_*Max*_ for *I*_*P*_ was small compared to *I*_*W*_. The differences in *A*_*Max*_ due to different country for both ideotypes were small. The mean ‘stay green’ trait (*S*_*G*_) in *I*_*W*_ was 73–84% greater than *C*_*L*_ in the UK and NZ (Table 1). Mean *S*_*G*_ in *I*_*P*_ was 21% smaller than *C*_*L*_ in the UK, but 102% greater in NZ. On an average, *S*_*G*_ in *I*_*W*_ was 6% greater in NZ than the UK, whereas 155% greater for *I*_*P*_ in NZ than the UK. Improved canopy architecture of both the ideotypes, in terms of greater *A*_*Max*_ and *S*_*G*_, is one reason for their higher yield potentials. Larger *A*_*Max*_ helps in increasing intercepted solar radiation and photosynthesis, considered as important traits for high yield potential^18,32^. The *S*_*G*_ is another important trait which helps increasing grain yield under both water-limited and irrigated condition by delaying leaf senescence and increasing plant capacity to maintain active photosynthetic tissues longer during grain filling^44,45^. Larger number of grains per ear, greater average grain weight and high yield were reported for different crop cultivars, including wheat, with the improved stay green trait^46,47^. Past increases in yield potential of wheat have largely resulted from improvements in harvest index (HI) rather than increased biomass^6^. Further large increases in HI are unlikely, but an opportunity exists for increasing productive biomass and harvestable grain yield. Photosynthetic capacity and efficiency are bottlenecks to raising productivity and there is strong evidence that increasing photosynthesis will increase crop yields provided that other constraints do not become limiting^32^. Even small increases in the rate of net photosynthesis can translate into large increases in biomass and hence yield, since carbon assimilation is integrated over the entire growing season and crop canopy. Different review studies^7,32^ discussed the strategies to increase photosynthesis that are being proposed by the wheat yield consortium in order to increase wheat yields include selection for photosynthetic capacity and efficiency, maximize canopy light interception and photosynthesis by optimizing canopy architecture, increasing cumulative photosynthesis duration by improving the stay-green trait^7,32^.

**Table 1:** Cultivar parameters of locally adapted winter wheat *cv*.

Location	Country	Cultivar parameter^†^						
		*P*_*h*_ (°C day)	*P*_*p*_ (Leaf h^−1^day length)	*G*_*f*_ (°C day)	*A*_*max*_ (m^2^ leaf m^−2^ soil)	*S*_*G*_ (−)	*R*_*u*_ (%)	*W*_*ss*_ (−)
**Current winter wheat** ***cv*** **. Claire (** ***C***_***L***_ **)**								
All	UK & NZ	110.0	0.5	650.0	0.007	0.5	3.0	1.27
**Wheat ideotype designed under water limited condition (** ***I***_***W***_ **)**								
Edinburgh	UK	139.9	0.0800	897.9	0.75 × 10^−2^	0.5400	4.20	1.0860
Leeds	UK	140.0	0.0750	900.0	0.90 × 10^–2^	1.1300	6.10	1.3900
Rothamsted	UK	139.7	0.0580	900.0	0.79 × 10^−2^	0.9300	4.50	1.2440
Gore	NZ	140.0	0.1140	843.6	0.62 × 10^−2^	1.1400	5.10	1.2140
Lincoln	NZ	140.0	0.0500	900.0	0.77 × 10^−2^	1.0500	4.90	1.0000
Pukekohe	NZ	140.0	0.6720	899.7	0.82 × 10^−2^	0.5700	4.90	1.5890
**Wheat ideotype designed under potential condition (** ***I***_***P***_ **)**								
Edinburgh	UK	140.0	0.0510	897.4	0.79 × 10^−2^	0.6500	2.40	n/a
Leeds	UK	140.0	0.0590	900.0	0.74 × 10^−2^	0.1800	1.90	n/a
Rothamsted	UK	140.0	0.0530	890.1	0.79 × 10^−2^	0.3600	3.70	n/a
Gore	NZ	140.0	0.1330	825.3	0.66 × 10^−2^	1.2200	4.50	n/a
Lincoln	NZ	139.9	0.3450	889.3	1.00 × 10^−2^	0.5500	6.90	n/a
Pukekohe	NZ	139.8	0.8010	771.2	0.82 × 10^−2^	1.2600	3.20	n/a

Claire (*C*_*L*_), and wheat ideotypes optimized under current climate in water-limited (*I*_*W*_) and potential (*I*_*P*_) conditions in the United Kingdom (UK) and New Zealand (NZ).

*P*_*h*_: Phyllochron, *S*_*G*_: Stay green.

*P*_*p*_: Day length response, *R*_*u*_: Rate of root water uptake.

*G*_*f*_: Duration of grain filling, *W*_*ss*_: Maximum acceleration of leaf senescence due to H_2_O stress.

*A*_*max*_: Maximum area of flag leaf, *n/a*:*W*_*ss*_ is not applicable in potential condition (no water stress).

#### Phenology

**Figure 2:**
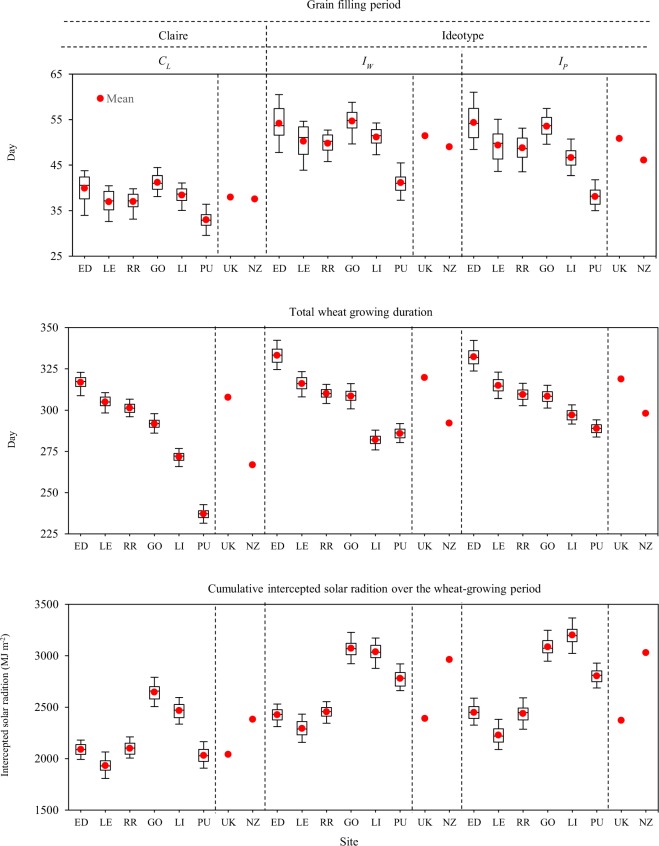
Grain filling period, total wheat growing duration and cumulative intercepted solar radiation over the wheat growing period of locally adapted winter wheat *cv*. Claire (*C*_*L*_) under current climate, and wheat ideotypes optimized under current climate in water-limited (*I*_*W*_) and potential (*I*_*P*_) conditions in the United Kingdom (UK) and New Zealand (NZ).

Mean grain filling duration of *C*_*L*_ was 38-days both in the UK and NZ. Mean grain filling duration for ideotypes were extended by 12–14 days for *I*_*W*_ and 9–13 days for *I*_*P*_ compared to *C*_*L*_ (Fig. 2). The mean anthesis date remained the same for both the ideotypes compared to *C*_*L*_ in the UK, but shifted forward by 15- and 24-days for *I*_*W*_ and *I*_*P*_, respectively in NZ (Supplementary Table S1). Averaged maturity or total crop-duration of *I*_*W*_ was extended by 12- and 25-days compared to *C*_*L*_ in the UK and NZ, respectively (Fig. 2 and Supplementary Table S1). Mean maturity of *I*_*P*_ was almost same as of *I*_*W*_ in the UK, but extended further by 6-days in NZ. The corresponding changes in phyllochron (*P*_*h*_) and mean day length response (*P*_*p*_) for both the ideotypes could be found in Table 1. Optimized phenology of the wheat ideotypes designed for the local climate is another reason for their high yield potentials. Optimal anthesis date is important for avoiding abiotic stresses, such as drought and high temperature, during reproductive period, resulting maximum yield through high number of grain set at anthesis^48,49^. Duration of the grain filling period is one of the important traits for increasing wheat yield potentials^50,51^. Extended grain filling period increases grain yield by not only increasing post anthesis light interception, photosynthesis and carbohydrate production translocated directly into developing grains, but also increasing the possibility of completion of re-translocation of labile carbohydrate mainly reserved in stem to the grains^23,52^.

#### Intercepted radiation, plant biomass and harvest index

The mean cumulative intercepted radiation over the entire wheat growing period of *I*_*W*_ exceeded by 17 and 25% over *C*_*L*_ in the UK and NZ, respectively (Fig. 2). Total intercepted radiations for *I*_*W*_ and *I*_*P*_ were 24 and 28% larger in NZ than the UK. Mean total plant biomass of the ideotype *I*_*W*_ was 27 and 37% higher compared to *C*_*L*_ in the UK and NZ, respectively (Fig. 1). Averaged plant biomass of *I*_*P*_ increased further by 2.3 and 5.2% compared to *I*_*W*_. The mean total plant biomasses for *I*_*W*_ and *I*_*P*_ were 28 and 31% greater in NZ than in the UK. Optimized canopy architecture and improved phenology maximized intercepted radiation for both ideotypes. Greater crop biomass had been reported resulting from higher intercepted solar radiation^6,53^. Rate of root water uptake (*R*_*u*_) also increased in accordance with increased plant biomass (Table 1). However, drought tolerance trait *W*_*ss*_ was not important for both idiotypes as winter wheat hardly faced drought stress under the current climate in the UK and NZ (Table 1). Additionally, sensitivity to heat or drought stress around flowering had very little effect on grain yield in the UK and NZ. Greater plant biomass increases the availability of assimilates for ear and grain development, resulting high yield potentials^6,18^. Mean HI increased slightly (3–10%, HI~0.50) (Fig. 1) for both the ideotypes compared to *C*_*L*_ in both countries as an indirect effect of optimization of different cultivar traits linked to crop canopy and phenology, such as *A*_*Max*_, *S*_*G*_ and grain filling period.

**Figure 3:**
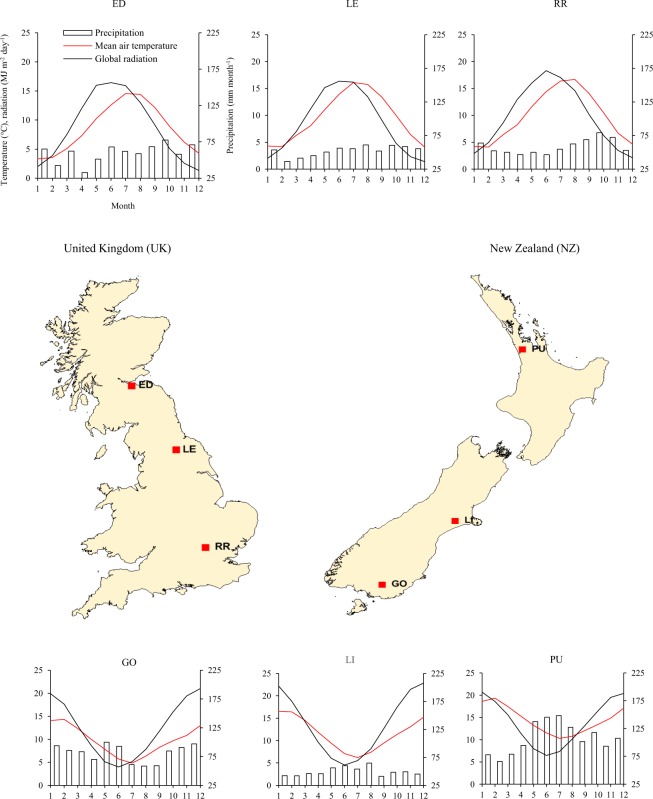
Location of six study sites across the United Kingdom (UK) and New Zeeland (NZ). ED: Edinburgh (UK), LE: Leeds (UK), RR: Rothamsted (UK), GO: Gore (NZ), LI: Lincoln (NZ), PU: Pukekohe (NZ). The average current climate (1981–2010) *viz*. mean air temperature, mean monthly precipitation and mean daily global radiation. Note that UK (north-hemisphere) and NZ (south-hemisphere) are in opposite hemisphere. Please note that MapInfo Pro v12.0 (https://www.pitneybowes.com/us/location-intelligence/geographic-information-systems/mapinfo-pro.html) was used to create these maps.

Overall, optimized canopy architecture and optimal phenology maximized intercepted solar radiation, biomass production, primary grain setting number and grain fill duration, resulting high yield potentials of wheat ideotypes (15–20 t ha^−1^) under water-limited and irrigated conditions in both countries. Although wheat ideotypes were designed and optimized for highest yield separately under water-limited and potential conditions, minor differences (3–4%) in yield potentials were observed between them due to minimum differences in optimized cultivar traits linked with canopy structure, phenology and root water uptake. Grater yield potentials (25–27%) were achieved in NZ than the UK for both ideotypes in water-limited and irrigated conditions. The main reasons for higher yield potential in NZ were relatively overall better optimized cultivar parameters and crop-traits in terms of canopy structure, phenology and root water uptake, and higher solar radiation, resulting greater intercepted cumulative radiation, biomass production and grain yield in NZ than the UK. Although mean wheat growing periods of both the ideotypes were shorter in NZ than the UK, higher solar radiation in NZ helps in greater cumulative intercepted radiation in NZ. Greater mean annual solar radiation (48%) across our study sites in NZ (southern hemisphere, 37–46°S) compared to the UK (northern hemisphere, 52–56°N) could be explained by lower latitude ( ≤ 10°) (Table 2), thinner O_3_ layer and lower atmospheric pollution in NZ than in the UK, and the asymmetric elliptical shape of the earth’s orbit, which brings the southern hemisphere closer to the sun during the southern summer than the northern hemisphere during the northern summer^54^. A positive relationship has been reported between solar radiation and wheat yield, whereas a negative relationship has been found between wheat yield and atmospheric pollution^55–57^. Another reason of high yield potential in NZ was higher photo-thermal-quotient in NZ than the UK. Although averaged annual air temperature was 2.7 °C greater in NZ than in the UK, the photothermal quotient (solar radiation/air temperature) was 18% greater in NZ than in the UK (Fig. 3 and Supplementary Fig. S1). Total crop biomass and grain yield were found to increase with an increasing photothermal quotient^52,58^. Higher mean annual precipitation in NZ (40%) than the UK could also be other reason of high yield potential in NZ, particularly under water limited condition for *I*_*W*_. The greater yield potential and field grain yields of winter wheat in NZ than in the UK have been reported by various studies^17,42,59–61^.

**Table 2:** Characteristics of the study sites representing major wheat growing regions across the United Kingdom (UK) and New Zealand (NZ).

ID	Site	Country^†^	Latitude (°)	Longitude (°)	Air temperature^††^ (°C)	Precipitation^††^ (mm yr^−1^)	Global radiation^††^ (MJ m^−2^ day^−1^)
ED	Edinburgh	UK	55.94	−3.31	8.6	717	8.7
LE	Leeds	UK	54.30	−1.53	9.5	626	8.6
RR	Rothamsted	UK	51.80	−0.35	9.8	700	9.8
GO	Gore	NZ	−46.12	168.89	9.8	976	12.4
LI	Lincoln	NZ	−43.70	172.00	11.6	596	13.6
PU	Pukekohe	NZ	−37.21	174.86	14.5	1296	14.0

^†^Note that UK (United Kingdom) and NZ (New Zealand) are in the north and south hemisphere, respectively.

^††^Mean current climatic conditions for period 1981–2010.

### Wheat yield potential and yield gap

The mean simulated management-optimal yield of winter wheat *cv*. Claire (*C*_*L*_) under current climate was 10.8 and 13.2 t ha^−1^ in the UK and NZ respectively (Fig. 1). The mean wheat yields simulated by Sirius in our present study are 39–57% greater than the national averages of the UK and NZ^17,62^. This could be explained by our assumptions about optimum agronomic management practices for effectively meeting N demand and achieving effective biological controls for any weed, disease and pest infestations, factors that generally reduce farmers’ as well as the national yields. However, mean current wheat yields of the present study are close to good year wheat yields (8–14 t ha^−1^) as reported by various studies across the UK and NZ^42,59,61,63,64^. Management optimal wheat yield potentials had been estimated and reported in the range of 7–13 t ha^−1^ in the north-western Europe including the UK mainly due to favourable climatic conditions and adapted local cultivars^15,19^.

The simulated yields of Claire (*C*_*L*_) and potential yields of wheat ideotypes (*I*_*W*_ and *I*_*P*_) under the current climate indicate the current mean yield gaps (Y_G_) of 4.2 and 5.8 t ha^−1^ in the UK and NZ, respectively in the water-limited or rainfed condition, and 4.7 and 6.2 t ha^−1^ in the UK and NZ, respectively in the potential or irrigated condition (Fig. 1). Mean yield gaps in the UK and NZ represent 28 and 31% of the yield potentials, respectively under water-limited condition. On the other hand, mean yield gaps under the potential condition represent 30 and 32% of the potential yields in the UK and NZ, respectively (Fig. 1). Yield gap variance due to sites was low (0.01–0.14 t ha^−1^) in the UK, whereas yield gap variance was greater (0.39–0.62 t ha^−1^) in NZ. Mean yield gap increased by 9–13% under potential condition than the water-limited condition in the UK and NZ. On average, yield gaps are 38 and 32% bigger in NZ than the UK in water limited and potential condition, respectively.

The general yield gap, which is conventionally estimated as the difference between management optimized crop yield and farmer average yield, mainly exists due the poor or sub-optimal managements^9,16,65^. This management optimal yield gap could be narrowed down by improving or optimizing crop management practices. This yield gaps in cereals including wheat reported and reviewed by different researchers of around 1–5 t ha^−1^, representing 10~70% of management optimized yield of which about 80% is exploitable^8,9,11–13,15,19^. As managements are near optimum in developed countries at high latitude with high wheat productivity, such as UK, NZ, further yield gain due to the improvements in managements is limited for current cultivars. Thus, management optimal yield gap for the current wheat cultivars is smaller in these high productive countries^15,19^.

Ideotype optimization under the local climatic condition in our study indicates the possibilities of achieving a substantial improvement in yield potentials of wheat (4–6 t ha^−1^) compared to the current cultivar in the UK and NZ. The average yield gaps (Y_G_) in the UK and NZ are of 28–32% of the potential (ideotype) yields under water-limited and potential conditions. Greater mean yield potentials of both ideotypes (25–27%) in NZ compared to the UK resulted into higher yield gap (32–38%) in the NZ than the UK under both water-limited and potential conditions. These were driven by better optimized parameterization, higher solar radiation, photo-thermal-quotient and precipitation in NZ than the UK as discussed above. Yield gaps under potential condition in both countries are driven by local climatic conditions, except water-limitation. Whereas, water-limitation was an additional constraint under water-limited condition. These explain why yield gap was slightly greater under potential than the water-limited condition.

The yield gaps in the present study are resulted from the optimal combination of plant traits by tapping the natural genetic variation observed in wheat germplasm, resulting potential wheat improvements to exploit most of the local climatic conditions, such as radiation, photothermal-quotient, temperature and precipitation. Thus, the yield gap in the present study is based on better adaptation of wheat to local climatic and environmental conditions in addition to optimal management practices. We have optimized wheat ideotypes by using the full parameter ranges in a multidimensional space of cultivar parameters, considering the basis of crop physiology and within the range of the observed genetic variations (Table 3). Main possible impacts of long-term abiotic stresses on crop growth and yield and short-term abiotic stresses were accounted along with the possible trade-offs and interactions among different plant traits^27,28^. Substantial yield gaps of 4–6 t ha^−1^ still exist in the UK and NZ despite intensive efforts in wheat breeding programmes and near optimal crop-management practices in those high productive countries. The ideotypes designed in the present study could be used as a road map by plant scientists and breeders for wheat improvement and genetic adaptation for high yield potentials in the UK and NZ. Recent advances in annotated reference genome of wheat^66^ and modern plant breeding technologies (*e.g*., molecular-marker-assisted breeding, chemical and genetic modulation and gene-editing)^31,67^, the existence of large natural genetic variation in the target traits (Table 3) along with wheat ideotype designs in the present study could assist plant breeders for developing the desirable cultivars that take the advantage of local genetic yield potential^3,6,7,20^. It could be possible to exploit the present yield gaps (28–32% the potential) even in the high productive countries through crop improvement and genetic adaptation. Tapping the genetic yield potential and closing the yield gap would help in increasing wheat productivity and grain production towards food security^3,7,9,32^. The method of designing wheat ideotypes and the estimation of yield gap for a target environment described in the present study is generic in nature, and therefore it could be used globally. However, the extent of possible yield gap would depend on local climatic and environmental conditions and existing crop-management practices.

**Table 3:** Sirius cultivar parameters used for designing wheat ideotypes under the current local climatic conditions, and genetic variation observed in those parameters for wheat.

Parameters	Symbol	Unit	Range used in model optimization	Genetic variation	Reference
***Phenology***					
Phyllochron	*P*_*h*_	°C day	80–140	≤20%	Ishag *et al*. (1998)^76^; Mosaad *et al*. (1995)^77^
Day length response	*P*_*p*_	Leaf h^−1^day length	0.065–0.900	9.74–107.40^*^	Kosner and Zurkova (1996)^78^
Duration of grain filling	*G*_*f*_	°C day	500–900	≤40%	Akkaya *et al*. (2006)^72^; Charmet *et al*. (2005)^79^; Robert *et al*. (2001)^80^
***Canopy***					
Maximum area of flag leaf	*A*_*Max*_	m^2^ leaf m^−2^ soil	0.005–0.01	≤40%	Fischer *et al*. (1998)^81^; Shearman *et al*. (2005)^82^
Stay green	*S*_*G*_	—	0.00–1.50		
***Root water uptake***					
Rate of root water uptake	*R*_*u*_	%	1.0–5.0	Large variation	Asseng *et al*. (1998)^83^; Manschadi *et al*. (2006)^73^
***Drought tolerance***					
Maximum acceleration of leaf senescence due to water stress	*W*_*ss*_	—	1.0–1.7		

*Varietal difference in number of days till heading under long- and short-day conditions found between 9.74 and 107.40 in a photoperiodic response experiment (Kosner and Zurkova 1996).

## Methods

### Target sites

For the present study, two high wheat-productive countries were selected, *viz*. the United Kingdom (UK) in the northern hemisphere and New Zealand (NZ) in the southern hemisphere^17^. Three sites were selected across major wheat-growing regions in the UK, covering high (northern) (Edinburgh: ED) and medium (Leeds: LE) to low (southern) (Rothamsted: RR) latitudes (Fig. 3). Similarly, another three sites were selected across major wheat-growing regions in NZ, covering high (southern) (Gore: GO) and medium (Lincoln: LI) to low (northern) (Pukekohe: PU) latitudes. Figure 3 and Table 2 show the detailed site characteristics.

### Climate

A 30-years (1981–2010) of daily observed weather data at each study site was used for estimating site parameters for the local climate. To assess inter-annual variation in crop production, a 100 years of daily weather data at each site was generated by using a stochastic weather generator (*LARS-WG 6.0*)^68^ based on the observed local climate parameters, hereafter defined as the ‘baseline-climate’ or the ‘current climate’ at individual sites. The mean annual air temperature, annual precipitation and mean daily global radiation of the baseline-climate were 9.3 °C, 681 mm yr^−1^ and 9.0 MJ m^−2^ day^−1^, respectively in the UK, and 12 °C, 956 mm yr^−1^ and 13.3 MJ m^−2^ day^−1^, respectively in NZ (Fig. 3 and Table 2).

### Sirius model

Sirius (2018) is a process-based wheat simulation model with a daily timescale and with an optimization framework, which facilitates designing ideotypes and optimizing cultivar parameters for target environment. The model requires daily weather data, a cultivar description, a soil physical description and management information as model inputs. A detailed description of the Sirius model can be found elsewhere^27,28,33,37,52^. Briefly, Sirius consists of various sub-models that describe soil, plant phenological development, water and nitrogen (N) uptake, photosynthesis and biomass production, and the partitioning of photosynthates into leaf, stem, grain and root. Photosynthesis and biomass production are simulated on a daily basis as the product of intercepted photosynthetically active radiation (*PAR*) and radiation use efficiency (*RUE*), limited by temperature and water stress. The N limitation and water stress reduce photosynthesis and new biomass production. Crop development rate and maturity are governed by phyllochron (*P*_*h*_), day length response (*P*_*p*_) and duration of grain filling (*G*_*f*_). Phenological development is calculated from the mainstem leaf appearance rate and final leaf numbers, with the latter determined by responses to day length and vernalisation. Leaf senescence is expressed in thermal time and linked to the rank of the leaf in the canopy. Leaf senescence could be accelerated by N limitation, or by abiotic stress, *viz*. temperature or water stresses. Soil is described as a cascade of 5-cm layers up to a user-defined depth. In addition to simulating the impacts of common water-limitation on crop-growth and yield, Sirius also simulates the effects of drought and heat stresses during the reproductive development on grain number and size.

### Designing wheat ideotypes

In the present study, a crop ideotype was defined as a set of Sirius cultivar parameters that would deliver high yield performance in a target environment when optimized. We used *cv*. Claire (*C*_*L*_) as a ‘baseline’ cultivar; it is a popular winter wheat variety in Europe, including the UK, and NZ, for its soft milling, early sowing, lodging and disease-resistant characteristics, and consistent high yield performance^69,70^. Claire has been used extensively as a parent in many wheat breeding programmes^70^. We also used *cv*. Claire as a ‘parent’ for designing the wheat ideotypes in the present study. Ideotypes were designed for both rainfed or water-limited condition (*I*_*W*_) and irrigated or potential condition (*I*_*P*_).

### Target traits for designing wheat ideotype

A total of seven Sirius cultivar parameters related with different plant traits were selected to design wheat ideotypes due to their a) importance in improving yield, b) large natural variations observed in wheat germplasms, c) potential for improvement through genetic adaptation^22,51^. Preliminary model runs in the present study as well as our previous studies^27,28^ indicated that the sensitivity to heat or drought stress around flowering had very little effect on grain yield in the UK and NZ. For this reason, sensitivity or tolerance to heat and drought stress was not included in the list of target traits. The targeted cultivar parameters and traits are summarized in Table 3 and described briefly as below.

#### Canopy

The potential maximum area of flag leaf (*A*_*Max*_) is a key trait in modifying the rate of canopy expansion and the maximum achievable leaf area index (LAI), which in turn will change the pattern of light interception and transpiration and, therefore, affect crop growth and final grain yield^22,51^. Delaying leaf senescence after anthesis is a possible strategy to increase grain yield by extending the duration of leaf senescence and maintaining the green leaf area longer: the so-called ‘stay green’ trait (*S*_*G*_)^44,45^. A larger value of *S*_*G*_ will delay leaf senescence and maintain green leaf longer for photosynthesis after anthesis.

#### Phenology

The phyllochron (*P*_*h*_), daylength response (*P*_*p*_) and duration of grain filling (*G*_*f*_) are important phenological traits which could be modified to maximize grain yield under a target environment by optimizing the rate of crop development, duration of grain-filling, and timing of anthesis and maturity^50,71,72^. Increasing *G*_*f*_ will increase the amount of radiation intercepted and grain yield. Whereas, decreasing *G*_*f*_ due to water or heat stress will decrease the grain yield because not only for the reduction in intercepted radiation, but also by limitation on labile carbohydrate translocation to the grain due to time shortage^23,52^.

#### Root water uptake

In Sirius, only a proportion of available soil water can be extracted from each layer in the root zone by the plant on any day, depending on water extraction efficiency (λ) and rate of root water uptake (*R*_*u*_). Faster root water uptake could reduce the current water stress experienced by plant, but could be risky under terminal drought. In contrast, an alternative strategy of slower root water uptake might increase yield by conserving water for successful completion of the life cycle in dry environments^73^.

#### Drought tolerance

The rate of leaf senescence increases under water stress due to the modification in daily increment of thermal time by a factor termed maximum acceleration of leaf senescence (*W*_*ss*_). Earlier leaf senescence will reduce grain yield due to reduction in intercepted radiation and photosynthesis and also reduction in translocation of the labile plant reserve carbohydrate to the grain due to premature termination of grain filling^23,51^. Under water stress, a higher value of *W*_*ss*_ will result in accelerated leaf senescence. On the other hand, *W*_*ss*_ is not important under potential condition (no water limitation).

### Ideotype optimization

An evolutionary search algorithm with self-adaptation (EASA) was used in Sirius to optimize both wheat ideotypes (*I*_*W*_ and *I*_*P*_) in a multidimensional parameter space with a complex fitness function to maximize wheat yield under the current climate at each site^34,74^. In each step of optimization, 16 new candidate ideotypes were generated from a ‘parent’ by perturbing its cultivar parameters randomly within the predefined parameters’ ranges as defined in Table 3. For each new candidate, yields were simulated for 100 years of the baseline-climate. Candidates with a coefficient of variation (*CV*) of yield exceeding 10% and a HI over 0.64 were removed from the selection process. A *CV* of less than 10% guarantees high yield stability, which is a desirable trait in crop cultivars, while the upper limit of HI was reported as 0.64^48^. The candidate with the highest mean yield was selected as a parent for the next step. The optimization process continued until no further improvement in yield potential was possible, or parameters converged to an optimal condition. To avoid local convergence and to explore fully the parameter spaces, we initialized the universal search optimization algorithm (EASA) with multiple-parents randomly scattered in the parameter space, except one parent that has the same cultivar parameters as Claire. A total of eight parents were used for each site. Convergences of cultivar parameters were robust in the UK and NZ, and a further increase in number of parents would have little benefits. For other regions with more extreme climates, the number of parents could be as high as 25. For each of the initial parents, EASA converges to an optimal combination of parameters; the best was selected as an optimal ideotype for a selected site.

### Estimation of yield gap by designing ideotype

The yield gap (Y_G_) of wheat in a given location was estimated as -

$${{\rm{Y}}}_{{\rm{G}}}={{\rm{Y}}}_{{\rm{GP}}}-{{\rm{Y}}}_{{\rm{M}}}$$

where, Y_GP_ is the ‘genetic’ yield potential in a local environment under irrigated or non-irrigated conditions, and Y_M_ is the management-optimal yield potential of a locally adapted current cultivar. Y_GP_ was estimated by optimizing physiological traits of wheat ideotypes^6,20–25^ both under non-irrigated (rainfed or water-limited) (*I*_*W*_) and irrigated (potential) conditions (*I*_*P*_) using Sirius crop model, as described in the ‘Designing wheat ideotypes’ and ‘Ideotype optimization’ sections. Y_M_ for the locally adapted winter wheat *cv*. Claire (*C*_*L*_), as mentioned in the ‘Designing wheat ideotypes’ section, was simulated using the same Sirius model assuming optimal management practices e.g., no N limitation and no yield losses due to any biotic stress such as disease, pest, weed etc.

### Simulation setup

We used Sirius version 2018 (available from https://sites.google.com/view/sirius-wheat). A single soil-water profile, *Rothamsted*, with a total available water capacity of 210 mm, was used for all sites in the UK, and a single soil-water profile, *Lincoln*, with a total available water capacity of 270 mm, was used for all sites in NZ, to eliminate site-specific soil effects from the analysis. Typical local sowing dates of 20-October in the UK and 20-April in NZ were used. For designing ideotype, a 10% increase in light use efficiency (*LUE*) was used^75^. Zhu *et al*.^75^ showed that up to 10% more carbon would be assimilated if the Rubisco specificity factor (λ) that represents the discrimination between CO_2_ and O_2_, is optimal under the current atmospheric CO_2_ level. Model parametrisation was the same for heat stress around flowering as in Stratonovitch and Semenov^27^ and drought stress around flowering as in Senapati *et al*.^28^. In all the model simulation, we assumed optimal agronomic managements, *e.g*. no N limitation or yield losses due to disease, pests or competition with weeds.

## Data Availability

Sirius version 2018 used in the present study is available from https://sites.google.com/view/sirius-wheat. The stochastic weather generator *LARS-WG 6.0* is available from https://sites.google.com/view/lars-wg. All data generated or analysed during this study are included in this published article and its Supplementary Information file. Any further information regarding the current study is available from the corresponding author on reasonable request.

## Supplementary information

Supplementary Information
