# Supplementary material for: Assessing yield gap in high productive countries by designing wheat ideotypes
Source: Sci Rep. 2019 Apr 2;9:5516. doi: 10.1038/s41598-019-40981-0 (PMC6445095; doi:10.1038/s41598-019-40981-0)
Supplement: Supplementary file 1 — Supplementary Information [file 41598_2019_40981_MOESM1_ESM.pdf]

Assessing yield gap in high productive countries by designing wheat ideotypes

Nimai Senapati* & Mikhail A. Semenov

Department of Plant Sciences, Rothamsted Research, West Common, Harpenden, Herts, AL5 2JQ, United Kingdom

**Corresponding author*

Email address: nimai.senapati@rothamsted.ac.uk; nimaisenapati@gmail.com;

Supplementary Information

Supplementary Table S1. Sowing, anthesis and maturity dates of locally adapted winter wheat *cv.* Claire (C_L) under current-climate, and wheat ideotypes optimized under current-climate in water-limited (I_W) and potential (I_P) conditions at selected study sites representing major wheat growing regions across the United Kingdom (UK) and New Zealand (NZ)

Site†	Sowing	Anthesis		Maturity	
		Mean	SD (day)	Mean	SD (day)
		C_L			
ED (UK)	20-October	2-July	2.1	1-September	4.2
LE (UK)	20-October	25-June	2.3	21-August	3.6
RR (UK)	20-October	22-June	2.9	17-August	3.5
GO (NZ)	20-April	4-December	2.3	5-February	3.5
LI (NZ)	20-April	19-November	2.7	16-January	3.3
PU (NZ)	20-April	21-October	3.1	13-December	3.6
		I_W			
ED (UK)	20-October	3-July	2.6	18-September	6.3
LE (UK)	20-October	23-June	2.7	1-September	4.9
RR (UK)	20-October	18-June	2.9	25-August	3.6
GO (NZ)	20-April	8-December	2.5	22-February	4.3
LI (NZ)	20-April	18-November	2.8	27-January	3.5
PU (NZ)	20-April	3-December	2.7	30-January	3.7
		I_P			
ED (UK)	20-October	2-July	2.7	17-September	6.1
LE (UK)	20-October	22-June	2.8	30-August	5.0
RR (UK)	20-October	18-June	2.9	25-August	4.0
GO (NZ)	20-April	10-December	2.4	22-February	4.1
LI (NZ)	20-April	7-December	2.5	11-February	3.6
PU (NZ)	20-April	10-December	2.7	2-February	3.3

ED (UK): Edinburgh, UK
LE (UK): Leeds, UK
RR (NZ): Rothamsted, UK
GO (NZ): Gore, NZ
LI (NZ): Lincoln, NZ
PU (NZ): Pukekohe, NZ

Mean: mean over 100 years
SD: Standard deviation over 100 years
†Note that UK (United Kingdom) and NZ (New Zealand) are in the north and south hemisphere, respectively

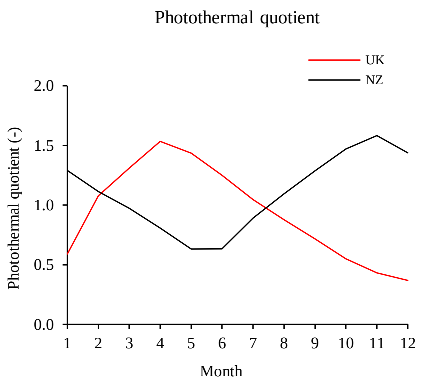

Supplementary Figure S1. Mean current photothermal quotient (solar radiation/air temperature) over the 12 months period across the study sites representing major wheat growing regions in the United Kingdom (UK) and New Zealand (NZ).
